# Changes in SedLine-derived processed electroencephalographic parameters during hypothermia in patients undergoing cardiac surgery with cardiopulmonary bypass

**DOI:** 10.3389/fcvm.2023.1084426

**Published:** 2023-07-04

**Authors:** Alessandro Belletti, Dong-Kyu Lee, Fumitaka Yanase, Thummaporn Naorungroj, Glenn M. Eastwood, Rinaldo Bellomo, Laurence Weinberg

**Affiliations:** ^1^ Department of Intensive Care, Austin Hospital, Heidelberg, VIC, Australia; ^2^ Department of Anaesthesia and Intensive Care, IRCCS San Raffaele Scientific Institute, Milan, Italy; ^3^ Department of Anesthesiology and Pain Medicine, Dongguk University Ilsan Hospital, Goyang, Republic of Korea; ^4^ Australian and New Zealand Intensive Care Research Centre, Monash University, Melbourne, VIC, Australia; ^5^ Department of Intensive Care, Siriraj Hospital, Mahidol University, Bangkok, Thailand; ^6^ Department of Critical Care, The University of Melbourne, Melbourne, VIC, Australia; ^7^ Data Analytics Research and Evaluation Centre, The University of Melbourne and The Austin Hospital, Melbourne, VIC, Australia; ^8^ Department of Anaesthesia, Austin Hospital, Heidelberg, VIC, Australia

**Keywords:** anesthesia, electroencephalography, cardiac surgery, neuromonitoring, neuroprotection, propofol, delirium, cardiopulmonary bypass

## Abstract

**Objective:**

Processed electroencephalography (pEEG) is used to monitor depth-of-anesthesia during cardiopulmonary bypass (CPB). The SedLine device has been recently introduced for pEEG monitoring. However, the effect of hypothermia on its parameters during CPB is unknown. Accordingly, we aimed to investigate temperature-induced changes in SedLine-derived pEEG parameters during CPB.

**Design:**

Prospective observational study.

**Setting:**

Cardiac surgery operating theatre.

**Participants:**

28 patients undergoing elective cardiac surgery with CPB.

**Interventions:**

We continuously measured patient state index (PSI), suppression ratio (SR), bilateral spectral edge frequency (SEF) and temperature. We used linear mixed modelling with fixed and random effects to study the interactions between pEEG parameters and core temperature.

**Measurements and main results:**

During CPB maintenance, the median temperature was 32.1°C [interquartile range (IQR): 29.8–33.6] at the end of cooling and 32.8°C (IQR: 30.1–34.0) at rewarming initiation. For each degree Celsius change in temperature during cooling and rewarming the PSI either decreased by 0.8 points [95% confidence interval (CI): 0.7–1.0; *p* < 0.001] or increased by 0.7 points (95% CI: 0.6–0.8; *p* < 0.001). The SR increased by 2.9 (95% CI: 2.3–3.4); *p* < 0.001) during cooling and decreased by 2.2 (95% CI: 1.7–2.7; *p* < 0.001) during rewarming. Changes in the SEF were not related to changes in temperature.

**Conclusions:**

During hypothermic CPB, temperature changes led to concordant changes in the PSI. The SR increased during cooling and decreased during rewarming. Clinicians using SedLine for depth-of-anesthesia monitoring should be aware of these effects when interpreting the PSI and SR values.

## Introduction

Both excessive as well as insufficient anesthesia depth may be associated with worse perioperative outcomes. Excessive anesthesia depth may be associated with the development of delirium, hemodynamic instability, prolonged intensive care unit (ICU) stay and long-term cognitive dysfunction. Conversely, insufficient anesthesia may be associated with the development of delirium, post-traumatic stress disorders, excessive circulating catecholamines and, in worst-case scenarios, intraoperative awareness (1).

Accordingly, monitoring for adequate depth of anesthesia during procedures requiring neuromuscular blockade is recommended by current guidelines, especially when total intravenous anesthesia is used (2). In patients undergoing cardiac surgery, the risk of awareness is considered particularly possible high during cardiopulmonary bypass (CPB) because conventional clinical parameters used to assess depth of anesthesia (i.e., heart rate and blood pressure) are profoundly altered by CPB, aortic cross-clamp and induced cardiac arrest (3–5). This has made the use of depth-of-anesthesia monitoring desirable.

Several depth-of-anesthesia monitoring devices are commercially available (6, 7). These devices obtain continuous, non-invasive processed electroencephalogram (pEEG) signals using adhesive gel electrodes placed on the forehead. The signal is subsequently amplified, filtered and processed through proprietary algorithms to provide a final index of anesthesia depth, as well as other indices such as burst suppression ratio (SR), electromyographic activity, and indices of signal quality (6, 8). Among such devices, the SedLine device (Masimo, Irvine, CA, USA) is one of the most recently introduced (7, 9). However, although some studies have shown that, in patients undergoing cardiac surgery, changes in temperature during CPB can independently affect the bispectral index (BIS) (10–13), there are no available data on the effect of temperature on SedLine-derived parameters.

Accordingly, we conducted a prospective observational study in patients undergoing cardiac surgery to investigate whether and to what extent changes in temperature during CPB affect SedLine-derived parameters.

## Methods

This was a single-centre, prospective, observational study performed on adult patients undergoing cardiac surgery requiring CPB in a teaching hospital in Australia. The study was approved by the institutional ethics committee (Austin Health Ethics Committee, Project No.: Audit/19/Austin/59). As the use of SedLine to monitor the depth of anesthesia was the standard of care in our institution, informed consent was waived because of the observational nature of the study.

### Inclusion and exclusion criteria

We included adult patients (i.e., over 18 years of age) who underwent elective cardiac surgery with planned use of CPB. We excluded patients if their procedure was planned to be off-pump, or if they had known baseline electroencephalography (EEG) alterations, pre-operative cognitive impairment or intellectual disability, a history of epilepsy, or a suspected or confirmed pregnancy.

### Anesthesia and cardiopulmonary bypass management protocol

All procedures were performed under general anesthesia. In all patients, monitoring included 5-lead electrocardiogram, pulse oximetry, invasive arterial blood pressure, central venous pressure, pulmonary artery pressure and cardiac index (obtained with a pulmonary artery catheter), body temperature (bladder temperature), diuresis and transesophageal echocardiography. Depth of anesthesia was monitored with SedLine in all patients. Regional cerebral oximetry was monitored at the discretion of the attending anesthesiologist. The anesthetic regimen was at the discretion of the attending anesthesiologist. However, in all cases, anesthesia was maintained with an intravenous continuous propofol infusion during CPB.

### Technology

Processed EEG was recorded using the Next Generation SedLine® Brain Function Monitoring device (Masimo, Irvine, CA, USA). The depth-of-anesthesia parameter displayed by SedLine is the Next Generation Patient State Index (PSI), a dimensionless index of sedation depth ranging from 0 to 100 (with 0 indicating a deeply sedated patient with an isoelectric EEG and 100 a fully awake patient), elaborated through a proprietary algorithm (7, 9). Additionally, the SedLine monitor displays two bilateral raw EEG traces, bilateral density spectral array and spectral edge frequency (SEF) (14), the burst suppression ratio (SR), the percentage of artefacts detected by the device as a measure of signal quality, and electromyographic (EMG) activity.

### Data collection and data cleaning

In all patients, SedLine monitoring was started on arrival in the operating theatre before the induction of anesthesia and then discontinued at skin closure before transferring the patient to the ICU. The SedLine device recorded all parameters at 2 s intervals. All SedLine-derived data were downloaded from the device immediately after surgery.

Temperature data obtained through the nasopharyngeal or bladder probe were automatically recorded every 20 s on the CPB machine software and downloaded at the end of the procedure. The CPB machine also automatically measured and recorded data on PaO_2_, PaCO_2_ and hemoglobin every 20 s. Additionally, one of the investigators manually recorded SedLine and temperature data on a case report form every 10 min to allow for a check of data accuracy.

In addition to SedLine and temperature data, baseline characteristics, the type of procedure, the duration of CPB and aortic cross-clamp, and outcome data were also recorded.

These data were subsequently merged into one electronic data sheet for each patient using Microsoft Excel, version 1911 (Microsoft, 2019, USA). According to the recorded event marks, the data on hypothermia induction, maintenance and recovery phases were separated and merged into another database. Before data analysis, all recordings were reviewed by two investigators to check for data accuracy and possible outliers. Using the scatter plots of each pEEG value against time or core temperature, a visual check was performed for artefact values with recorded artefact levels as reference. After manual trimming, all starting time points were set to zero. For the SR, all recorded values of zero were removed.

### Statistical analysis

Statistical analysis was performed using IBM SPSS Statistics for Windows, version 23 (IBM, 2015, Armonk, NY, USA) and R, version 3.6.1 (R Development Core Team, 2018, Vienna, Austria). A normality test was performed for continuous variables, and, when the normality assumption was violated, a non-parametric statistical method was applied. Descriptive statistics were used for demographic data presentation.

#### Linear mixed modelling with fixed and random effects

Considering general concepts concerning EEG and hypothermia, we presumed that, at a certain point, all pEEG values would converge into one point as the core temperature decreased (15). At the start of CPB, each patient had a various core temperature and their own pEEG value. The target temperature of hypothermia was also varied individually, as was the duration of hypothermia according to the type of surgery. Additionally, the patient's response to lowering their core temperature could vary.

Therefore, to evaluate the effect of hypothermia on the pEEG at the different hypothermia targets, we performed a visual check of each patient's pEEG response over the measured time points. We hypothesized that the response of the pEEG to the lowering, maintaining, or increasing of the patient's core temperature would follow random slopes (i.e., each patient would have their own response) and converge into one point (i.e., we presumed a fixed intercept where the core temperature was 0°C). With this assumption, and the results of a visual check for the linear relationship between the pEEG and the core temperature of each patient, we evaluated the linear mixed model with random slope–effects between the pEEG and the core temperature during hypothermia induction and recovery periods.

For hypothermia induction and recovery periods, the major variation was the core temperature, which was measured every 20 s. We prespecified a linear mixed model with random effects for the core temperature for both periods. During the maintenance period, the core temperature was maintained within a narrow range, and the main variation was the duration of hypothermia. To evaluate the effect of hypothermia duration on the pEEG for this period, we used a linear mixed model with random effects by time instead of core temperature.

Given the differences in the responses of individual patients and in the values of pEEGs at the beginning of hypothermia maintenance, the random effects were considered for both the slope and intercept for the linear mixed-effects model during the hypothermia maintenance period. Unstructured covariance was used for random effects. The whole model was evaluated for fitness with −2 restricted log-likelihood and Schwarz's Bayesian criterion. A Type III sum of squares was used for fixed-effects evaluation. The variances of covariance parameter estimates were also tested with the Wald *Z*-test to evaluate the random effect between subjects.

#### Association between PSI values and postoperative complications

PSI values were divided into three different categories: <25 (deep anaesthesia), 25–50 (optimal anaesthesia depth), and >50 (light anaesthesia), as recommended by manufacturer (7, 9). During CPB (from pump-on to pump-off), the relative duration of each PSI level (>50, 25–50, <25) was identified and expressed as percentage of time over the total CPB duration.

Spearman/Pearson correlation analysis was performed according to the characteristics of the outcome variables.

## Results

Between 11 June and 29 July 2019, a total of 54 patients underwent cardiac surgery with CPB. Of these, 21 were excluded due to the unavailability of research personnel to collect complete intraoperative data, one was excluded as an emergency case, two were excluded due to exclusion criteria, and two were excluded due to technical failure in data recording by the CPB machine. Therefore, the remaining 28 patients were included in the analysis.

### Characteristics of the participants

The median duration of analyses was 2,380 s (IQR: 1,645 s–3,365 s), 4,160 s (IQR: 1,790 s–6,155 s) and 1,520 s (IQR: 1,260 s–2,085 s) for the hypothermia induction, maintenance and recovery periods, respectively. The total included time was 74,000, 125,760 and 48,460 s for the induction, maintenance and recovery periods, respectively. Patients' baseline and procedural characteristics are summarized in Table 1.

**Table 1:** Patients’ characteristics.

Characteristic	Value
Age (years), Mdn (IQR)	72.3 (60.3–79.6)
Height (cm), Mdn (IQR)	167 (158–173)
Weight (kg), Mdn (IQR)	79 (67–91)
Female	8 (28.6)
Diabetes	12 (37.5)
Hypertension	25 (89.3)
Congestive heart failure	14 (50.0)
Ischemic heart disease	20 (71.4)
Dyslipidemia	17 (60.7)
Cerebrovascular disease	8 (28.6)
Peripheral vascular disease	5 (17.8)
Chronic liver disease	1 (3.5)
Chronic respiratory disease	3 (10.7)
Chronic kidney disease	7 (25.0)
Surgical procedure	
Aortic arch replacement + stenting	1 (3.5)
AVR	2 (7.1)
AVR (mini-invasive)	1 (3.5)
Bentall's procedure	1 (3.5)
CABG	12 (37.5)
CABG and AVR	6 (21.4)
CABG and MV repair	1 (3.5)
David's procedure	1 (3.5)
MV repair	1 (3.5)
MVR	1 (3.5)
MVR (mini-invasive)	1 (3.5)
Ross procedure	2 (7.1)
Cardiopulmonary bypass time (min), Mdn (IQR)	124 (99–201)
Cross-clamp time (min), Mdn (IQR)	99 (74–160)
Deep hypothermic circulatory arrest	1 (3.5)
Selective cerebral perfusion	1 (3.5)

*N* = 28. Values are given as *n* (%) unless otherwise specified. IQR, interquartile range; AVR, aortic valve replacement; CABG, coronary artery bypass grafting; MVR, mitral valve replacement.

The median core temperatures measured during CPB were 35.2°C (IQR: 34.7°C–35.6°C) and 32.7°C (IQ: 30.4°C–34.0°C) at the start and end of the hypothermia induction period, respectively (Wilcoxon signed-rank test, *p* < 0.001). During the maintenance period, the core temperatures were 32.1°C (IQR: 29.8°C–33.6°C) and 32.8°C (IQR: 30.1°C–34.0°C) at the start and end of the period, respectively (Wilcoxon signed-rank test, *p* < 0.001). Statistically, the core temperatures were significantly different between at the start and end of the maintenance period despite core temperatures being kept within 2.0°C during this period. For the recovery period, the core temperatures were 32.7°C (IQR: 30.4°C–33.9°C) and 36.5°C (IQR: 36.5°C–36.7°C) at the start and end of the period, respectively (Wilcoxon signed-rank test, *p* < 0.001). Data on PaO_2_, PaCO_2_, hemoglobin and anesthetic agents used during CPB are presented in Table 2.

**Table 2:** Anaesthesia and arterial blood Gas analysis results during cardiopulmonary bypass.

Variable	Value
PaO_2_ (mmHg), M ± SD	
Cooling	351.4 ± 71.5
Maintenance	354.5 ± 53.9
Rewarming	320.1 ± 61.3
PaCO_2_ (mmHg), M ± SD	
Cooling	39.1 ± 8.7
Maintenance	42.4 ± 4.6
Rewarming	46.1 ± 5.3
Hemoglobin (g/dl), M ± SD	
Cooling	9.4 ± 2.9
Maintenance	9.9 ± 1.8
Rewarming	9.8 ± 2.3
Anesthesia medications during CPB, *n* (%)	
Propofol continuous infusion	28 (100)
Isoflurane in CPB oxygenator	22 (78.6)
Fentanyl bolus	13 (46.4)
Fentanyl continuous infusion	9 (32.1)
Alfentanil continuous infusion	9 (32.1)
Remifentanil continuous infusion	1 (3.5)
Dexmedetomidine continuous infusion	1 (3.5)

CPB, cardiopulmonary bypass.

### Analysis of the processed electroencephalography

#### Hypothermia induction period

Details on changes in pEEG-derived parameters during hypothermia induction period are presented in Table 3. According to the random-slope linear mixed modelling, the PSI decreased 0.84 points with each degree Celsius core temperature drop [95% confidence interval (CI): 0.68–0.99; *p* < 0.001]. The SR increased by 2.9 (95% CI: 2.3–3.4; *p* < 0.001).

**Table 3:** Changes in processed electroencephalography-derived parameters during cooling and rewarming.

	Change per 1°C change in temperature	95% CI	*F* statistics	*p*-value
Cooling				
PSI	−0.84	−0.99 to −0.68	*F*(1, 193.4) = 111.1	<0.001
SR	2.9	2.3–3.4	*F*(1, 315.2) = 104.6	<0.001
SEF, R (Hz)	−0.18	−0.24 to −0.12	*F*(1, 683.7) = 34.7	<0.001
SEF, L (Hz)	−0.22	−0.28 to −0.16	*F*(1, 333.0) = 47.5	<0.001
Rewarming				
PSI	0.70	0.60–0.81	*F*(1, 27.1) = 194.4	<0.001
SR	−2.2	−2.7 to −1.7	*F*(1, 188.5) = 85.4	<0.001
SEF, R (Hz)	0.18	0.10–0.26	*F*(1, 210.2) = 19.3	<0.001
SEF, L (Hz)	0.28	0.21–0.36	*F*(1, 215.7) = 50.3	<0.001

CI, confidence interval; PSI, patient state index; SEF, R, spectral edge frequency, right cerebral hemisphere; SEF, L, spectral edge frequency, left cerebral hemisphere; SR, suppression ratio.

**Figure 1:**
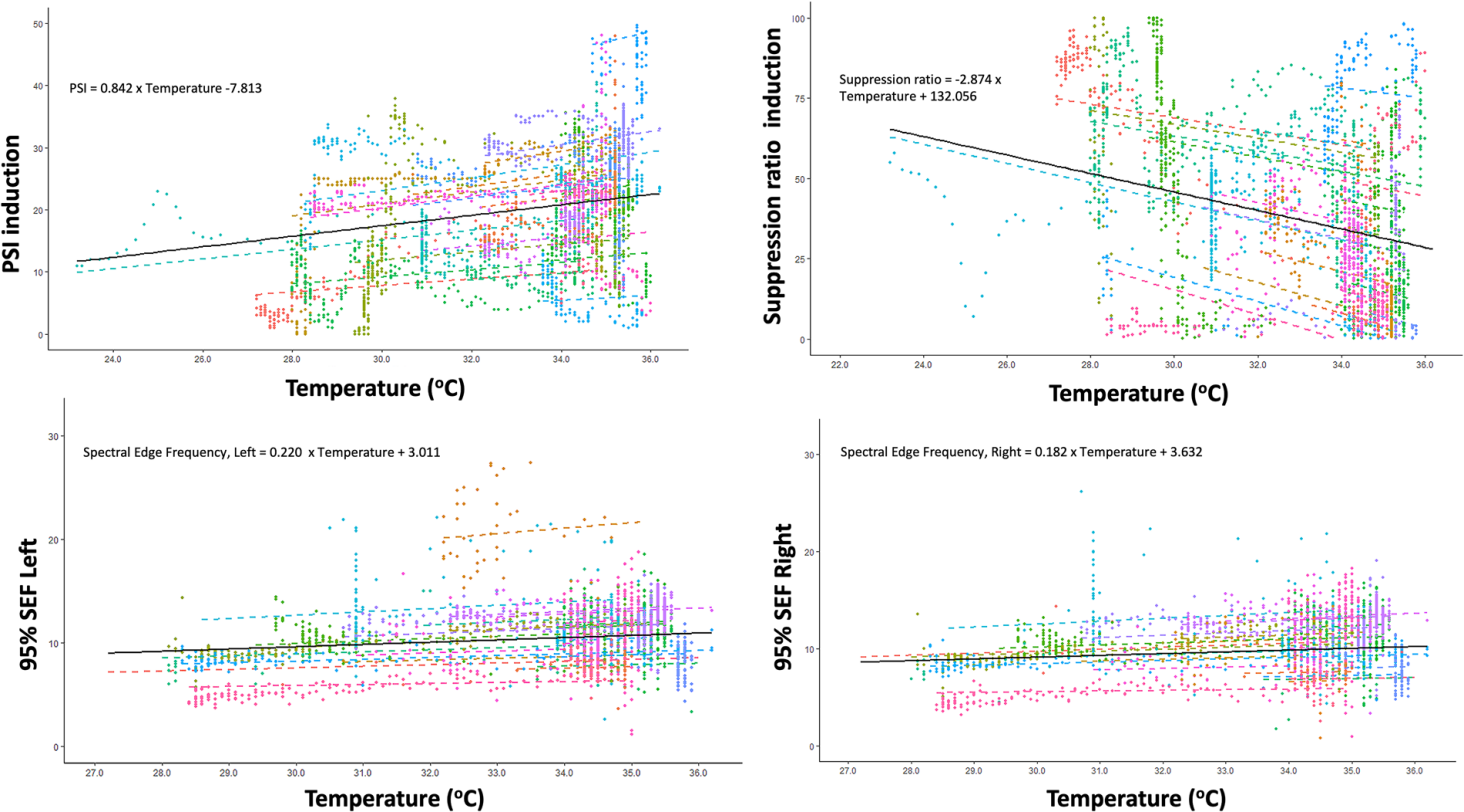
Correlation between changes in core temperature and changes in Patient State Index (PSI), Suppression ratio, Spectral Edge Frequency (SEF) of left brain hemisphere and SEF of right brain hemisphere during induction of hypothermia.

With lower temperatures, the SEF significantly decreased: by 0.18 Hz (95% CI: 0.12 Hz–0.24 Hz; *p* < 0.001), and by 0.22 Hz (95% CI: 0.16 Hz–0.28 Hz; *p* < 0.001), for the right and left, respectively (see Figure 1). Details on variation between patients are presented in the Supplementary Table S1.

#### Maintenance period

As previously described, the effects of hypothermia on the pEEG during the maintenance period were evaluated in relation to the duration of hypothermia. Details are presented in Table 4. The PSI had significant random variation at the beginning of hypothermia maintenance (*p* < 0.001); the random effect of hypothermia maintenance duration was not significant (*p* = 0.5). Considering this variability, the estimated linear relationship was not significant (*p* = 0.224). The estimated intercept was 21.3 (95% CI: 17.3–25.2; *p* < 0.001).

**Table 4:** Effect of hypothermia duration on processed electroencephalography-derived parameters during maintenance period.

EEG variable	Coefficient			Estimated variance of intercept		Estimated variance of duration	
	Coefficient	95% CI	*p*-value	Variance	*p*-value	Variance	*p*-value
PSI	5 × 10^−4^	−3 × 10^−4^ to 1.2 × 10^−3^	0.224	103.7	<0.001	2.2 × 10^−3^	0.500
SR	−4.1 × 10^−5^	−3 × 10^−3^ to 2.9 × 10^−3^	0.980	908.7	0.001	−6.3 × 10^−3^	0.890
SEF, R (Hz)	2.1 × 10^−4^	−3 × 10^−5^ to 4.4 × 10^−4^	0.077	15.2	0.001	2.0 × 10^−6^	0.996
SEF, L (Hz)	1.9 × 10^−4^	−2.3 × 10^−4^ to 5.9 × 10^−4^	0.346	21.4	0.001	−2.0 × 10^−3^	0.072

CI, confidence interval; PSI, patient state index; SEF, R, spectral edge frequency, right cerebral hemisphere; SEF, L, spectral edge frequency, left cerebral hemisphere; SR, suppression ratio.

The SR showed a significant random effect at the beginning of hypothermia maintenance (*p* = 0.001), and the duration of hypothermia maintenance was not a significant random effect (*p* = 0.891). Accepting that the intercepts were variable between patients, there was no significant linear relationship between the SR and the duration of hypothermia maintenance (*p* = 0.978).

The SEFs also had variability in the intercepts (*p* = 0.001 for both right and left), and the duration of hypothermia maintenance was not a significant random effect (right, *p* = 0.996; left, *p* = 0.072). During this period, there was no relationship between the right or left SEF and the duration of hypothermia maintenance (right, *p* = 0.077; left, *p* = 0.346). The estimated intercepts were 10.7 (95% CI: 9.1–12.3; *p* < 0.001), and 11.3 (95% CI: 9.4–13.2; *p* < 0.001) for the right and left, respectively.

#### Rewarming period

Details on changes in pEEG-derived parameters during rewarming period are presented in Table 3. During the rewarming period, every degree Celsius increase in core temperature was associated with an increase in the PSI by 0.70 (95% CI: 0.60–0.81, *p* < 0.001).

**Figure 2:**
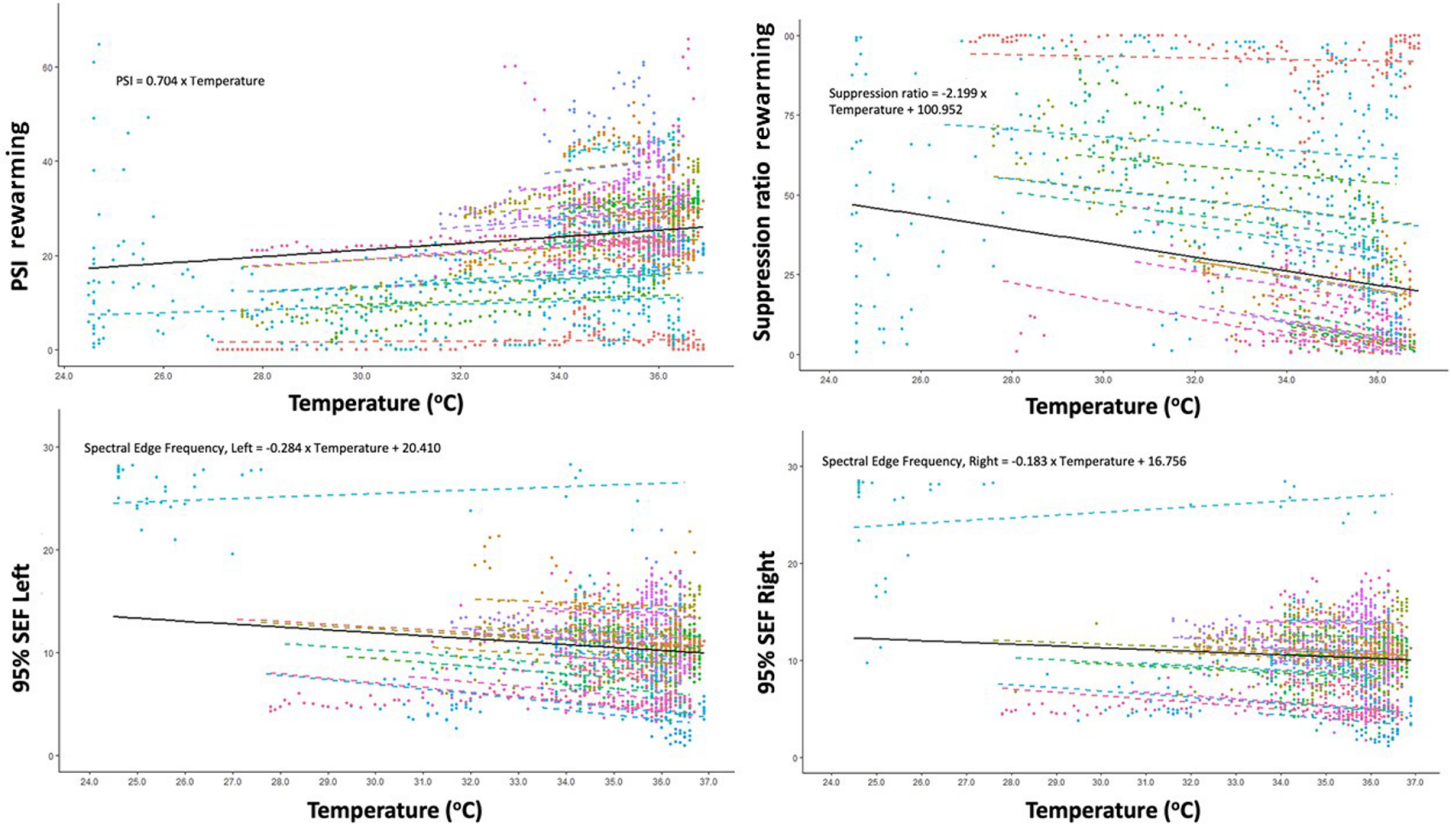
Correlation between changes in core temperature and changes in Patient State Index (PSI), Suppression ratio, Spectral Edge Frequency (SEF) of left brain hemisphere and SEF of right brain hemisphere during rewarming.

The SR decreased by 2.2 for each degree Celsius increase of the core temperature (95% CI: 1.7–2.7; *p* < 0.001). The SEF of both sides decreased according to increased core temperature. The right and left SEF were predicted to be, in response to increased core temperature of one unit, 0.18 Hz (95% CI: 0.10 Hz–0.26 Hz, *p* < 0.001) and 0.28 Hz (95% CI: 0.21 Hz–0.36 Hz, *p* < 0.001) for right and left, respectively (Figure 2). Details on variation between patients are presented in the Supplementary Table S1.

### Outcomes

Major complications are presented in Supplementary Table S2. A total of 13 patients (46%) required inotropic support in ICU, with three (10.7%) requiring inotropic support for >48 h. Acute kidney injury occurred in 14 patients (50%), with two patients requiring renal replacement therapy (7.1%). No patient had postoperative myocardial infarction. Delirium occurred in nine (32.1%) patients. Three patients (10.7%) required re-exploration for bleeding. One of them had a massive hemorrhage requiring immediate re-sternotomy at the bedside in ICU. The subsequent course was complicated by cardiogenic shock requiring extracorporeal membrane oxygenation, renal failure requiring renal replacement therapy and ischemic stroke. The patient died in ICU. There were no other in-hospital deaths.

Association between PSI levels and complications is presented in Supplementary Table S3. We found that exposure to a PSI level >50 during CPB was moderately associated with development of postoperative delirium, while no association with other complications was found.

## Discussion

### Key findings

In this single-center observational study, we quantified changes in the SedLine parameters in relation to changes in body temperature. We found that the PSI decreased by 0.84 points for each degree Celsius decrease in core temperature during the induction of hypothermia and increased by 0.70 points for each degree during the recovery phase. Similarly, SR increased during induction and decreased during the recovery period, while changes in the SEF were not related to changes in body temperature.

### Relationship to previous studies

Changes in EEG during hypothermia have been previously described in several studies. Early studies performed in the 50 s and 60 s showed that hypothermia has a suppressive effect on EEG (16, 17). More recently, since the introduction of pEEG monitoring in anesthesia, several studies have investigated the effect of hypothermia on the BIS (4), the most widely described pEEG parameter (7).

Overall, these studies found that hypothermia induced a decrease in the BIS of approximately 1 point per degree Celsius (11, 12, 18). A strong correlation between the BIS and temperature was also found in studies investigating cardiac surgery under deep hypothermic circulatory arrest (19, 20). A study comparing the BIS with entropy showed that the two indices have good correlation under normothermic conditions but poor agreement during hypothermia, suggesting that different depth-of-anesthesia indices may not be equivalent under hypothermic conditions (21).

In our study, we investigated, for the first time, the effect of hypothermia on the PSI, a novel depth-of-anesthesia index displayed by the SedLine monitor. Our results showed that changes in the PSI are comparable to those reported for the BIS (i.e., approximately 1 PSI point for each degree Celsius decrease). Interestingly, changes in the PSI were more pronounced during the induction of hypothermia than during rewarming. To the best of our knowledge, there have been no studies comparing changes in the PSI and the BIS during hypothermia. Thus, comparison between these two indices in this specific condition remains speculative.

In addition, we investigated changes in the SEF. Some studies have reported that the SEF remains relatively stable during hypothermia (22, 23), although such findings have not been consistent (3, 18, 24). Our results demonstrate the possible independence of the SEF from core temperature during hypothermia induction, maintenance and recovery periods. These findings imply that SEF variations during hypothermia may differ from PSI or SR responses to core temperature changes. These findings are consistent with previous reports that changes in the SEF are not consistent with BIS changes in the late phase of CPB (3) and during rewarming (24). Further, our findings confirm those from previous studies that have shown that EEG changes during the cooling phase of hypothermia differ from changes during rewarming (25, 26). However, it should be noted that absolute changes in the SEF in our study are unlikely to be clinically relevant (approximately 0.2 Hz for each degree Celsius; i.e., less than 1 Hz from 36 to 32°C) considering the wide range of SEF values (approximately 10–23 Hz) even in healthy volunteers (27).

### Study implications

The results of our study imply that SedLine-derived indices during CPB are influenced by changes in body temperature. Findings from our study further suggest that changes in body temperature have different effects on different SedLine-derived parameters. Our data suggest that the PSI correctly reflects EEG alterations during temperature changes and may be used to monitor the depth of anesthesia during hypothermic CPB. Furthermore, for the first time, we were able to provide an approximate estimation of the relative contribution of mild hypothermia to PSI changes: approximately 1 PSI point for each degree Celsius. Clinicians using pEEG monitoring during anesthesia or in the ICU should be aware of these changes when interpreting the values of pEEG indices and making clinical decisions.

Interestingly, we found that inadequately deep anesthesia was associated with development of postoperative delirium. The association between inadequate anesthesia depth (either too deep or to light) and delirium has already been described, and our findings are in line with this concept (1, 28), although our study was underpowered to investigate association between PSI levels and complications.

### Strengths and limitations

The strengths of our study include the continuous recording and analysis of temperature and pEEG data, unlike previous studies that had limited data collection and fixed time points (3, 21, 23). Furthermore, our data collection was not limited to the PSI but also included additional SedLine-derived parameters, such as the SEF and SR, and analysis their correlations.

Our study has several limitations. The anesthesia protocol was not mandatory; therefore, we cannot exclude the possibility of different effects of different anesthesia techniques on the pEEG-derived indices. However, our study reflects current clinical practice. Additionally, the study has all the limitations of a single-center trial. Accordingly, our findings may not be generalizable to other hospitals or to other non-cardiac surgery adult patients. We used only SedLine as depth-of-anaesthesia monitor. Accordingly, we were unable to make a direct comparison with other devices. Future studies should address whether different anesthetic regimens have different effects on SedLine-derived parameters during hypothermia induction, maintenance, and recovery. Furthermore, future studies should investigate whether different PSI-guided anesthesia regimens during hypothermia influence neurological outcomes after cardiac surgery with CPB. Our study provides baseline data that allow for the planning and design of such future studies.

## Conclusions

During hypothermic CPB using the SedLine device, temperature changes led to a decrease in the PSI with cooling and an increase with warming with changes of approximately 1 PSI point for each degree Celsius. The SR increased during cooling as the PSI decreased and decreased as the PSI increased during rewarming. In contrast, changes in the SEF were not related to changes in body temperature. Clinicians using SedLine for depth-of-anesthesia monitoring during CPB with induced hypothermia should be aware of these effects when interpreting the PSI and SR values.

## Data Availability

The raw data supporting the conclusions of this article will be made available by the authors, without undue reservation.
