# Supplementary material for: Changes in SedLine-derived processed electroencephalographic parameters during hypothermia in patients undergoing cardiac surgery with cardiopulmonary bypass
Source: Front Cardiovasc Med. 2023 Jul 4;10:1084426. doi: 10.3389/fcvm.2023.1084426 (PMC10352607; doi:10.3389/fcvm.2023.1084426)
Supplement: Supplementary file 1 [file Datasheet1.doc]

**SUPPLEMENTAL DIGITAL CONTENT**

**Changes in SedLine-derived processed electroencephalographic parameters during hypothermia in patients undergoing cardiac surgery with cardiopulmonary bypass**

**Index**

| 1. **Supplementary Table 1** | Page 2 |
| --- | --- |
| 1. **Supplementary Table 2** | Page 3 |
| 1. **Supplementary Table 3** | Page 4 |

**Supplementary Table 1.** Details on variation of processed electroencephalography-derived parameters between patients.

|  | **Inter-patient variation, %** | **Total estimated variance** | **Random effects for core temperature** | **Z-statistic** | **p-value** |
| --- | --- | --- | --- | --- | --- |
| Cooling |  |  |  |  |  |
| - PSI | 0.17 | 37.36 | 0.06 | 3.636 | *p* < 0.001 |
| - SR | 0.13 | 347.34 | 0.45 | 3.247 | *p* < 0.001 |
| - SEF, R - Hz | 0.11 | 3.86 | 0.004 | 3.530 | *p* < 0.001 |
| - SEF, L - Hz | 0.20 | 3.57 | 0.007 | 3.540 | *p* < 0.001 |
| Rewarming |  |  |  |  |  |
| - PSI | 0.18 | 39.39 | 0.07 | 3.651 | *p* < 0.001 |
| - SR | 0.13 | 327.6 | 0.4 | 3.299 | *p* = 0.001 |
| - SEF, R - Hz | 0.30 | 4.43 | 0.02 | 3.572 | *p* < 0.001 |
| - SEF, L - Hz | 0.40 | 4.17 | 0.02 | 3.640 | *p* < 0.001 |

PSI: patient state index; SEF, R: spectral edge frequency, right cerebral hemisphere; SEF, L: spectral edge frequency, left cerebral hemisphere; SR: suppression ratio

Supplementary Table 2: Outcomes

| **Outcome** | **Value** |
| --- | --- |
| Need for inotropes | 13 (46.4) |
| - Need for inotropes > 48 hours | 3 (10.7) |
| Myocardial infarction | 0 (0.0) |
| Acute kidney injury | 14 (50) |
| Renal replacement therapy | 2 (7.1) |
| Ischemic/hemorrhagic stroke | 1 (3.5) |
| Delirium | 9 (32.1) |
| Blood transfusion | 6 (21.4) |
| Surgical revision | 3 (10.7) |
| ICU readmission | 2 (7.1) |
| Length of ICU stay (hours), *Mdn* (IQR) | 35 (22–53) |
| Length of hospital stay (days), *Mdn* (IQR) | 7 (6–11) |
| In-hospital mortality | 1 (3.5) |

*Note*. *N* = 28. Values are given as *n* (%) unless otherwise specified. ICU = intensive care unit; IQR = interquartile range.

Supplementary Table 3: Association between PSI levels and postoperative complications

|  | **PSI > 50** | | **50 ≥ PSI > 25** | | **PSI ≤ 25** | |
| --- | --- | --- | --- | --- | --- | --- |
| **No.** | 5 (17.9%) | | 25 (89.3%) | | 27 (96.4%) | |
| duration% in corresponding patients | 4.2 (1.65‒16.275) [27.3:0.5] | | 51 (8.1‒76) [98.1:1.5] | | 62.2 (15.25‒82.45) [99.8:0.7] | |
| **Correlation** | | | | | | |
|  | Coefficient | P value | Coefficient | P value | coefficient | P value |
| Need for inotropes | -0.129 | 0.514 | 0.111 | 0.574 |  |  |
| Need for inotropes for > 48h | 0.11 | 0.579 | -0.15 | 0.446 | 0.179 | 0.363 |
| Myocardial infarction | NA | NA | NA | NA | NA | NA |
| Acute kidney injury | -0.172 | 0.38 | -0.009 | 0.964 | 0.084 | 0.671 |
| Renal replacement therapy | -0.143 | 0.466 | -0.052 | 0.795 | 0.103 | 0.602 |
| Stroke | -0.1 | 0.614 | -0.155 | 0.431 | 0.203 | 0.301 |
| Cerebral hemorrhage | NA | NA | NA | NA | NA | NA |
| Delirium | .435* | 0.021 | 0.081 | 0.684 | -0.213 | 0.276 |
| Blood transfusions | -0.038 | 0.85 | -0.086 | 0.663 | 0.048 | 0.806 |
| Surgical revision | -0.179 | 0.361 | -0.222 | 0.257 | 0.257 | 0.186 |
| ICU readmissions | -0.15 | 0.456 | 0.236 | 0.236 | -0.182 | 0.365 |
| Length of ICU stay (hours) | 0.062 | 0.753 | -0.32 | 0.097 | 0.315 | 0.102 |
| Length of hospital stay (days) | 0.085 | 0.667 | -0.101 | 0.608 | 0.093 | 0.639 |
| In-hospital mortality | -0.1 | 0.614 | -0.155 | 0.431 | 0.203 | 0.301 |

ICU = intensive care unit
